# Knowledge and Opinion on Cannabinoids Among Orthopaedic Traumatologists

**DOI:** 10.5435/JAAOSGlobal-D-21-00047

**Published:** 2021-04-19

**Authors:** Garwin Chin, Brent A. F. Etiz, Ariana M. Nelson, Philip K. Lim, John A. Scolaro

**Affiliations:** From the Department of Orthopaedic Surgery, University of California, Irvine, CA (Dr. Chin, Dr. Lim, and Dr. Scolaro), Orange, CA; the University of California, Irvine School of Medicine, Irvine, CA (Mr. Etiz); and the Department of Anesthesiology and Perioperative Care, Division of Pain Medicine, University of California (Dr. Nelson), Irvine, Orange, CA.

## Abstract

**Introduction:**

Cannabinoids are an increasingly popular therapy among orthopaedic patients for musculoskeletal conditions. A paucity of evidence to support their use in orthopaedics exists, likely because of the incongruence of federal and state legalization and the stigma surrounding cannabis. The purpose of this study is to elucidate sentiments and knowledge base of the orthopaedic trauma community with regard to cannabinoid-containing compounds.

**Methods:**

A 21-question online survey was distributed to the members of the Orthopaedic Trauma Association with a response window of 3 months.

**Results:**

We evaluated 251 responses. Most (88%) of the respondents did not believe that they were knowledgeable about the mechanism of action of cannabis/cannabidiol (CBD) but did feel that cannabis or CBD products play a role in managing postoperative pain (73%). Most respondents did not believe that they would be stigmatized if they suggested CBD (83%) or cannabis (67%) to patients. Despite this, fewer respondents have suggested CBD (38%) or cannabis (29%) to their patients.

**Conclusions:**

Sentiment toward cannabinoids among orthopaedic traumatologists is remarkably favorable; however, in-depth understanding is admittedly poor and routine use is uncommon. More clinical research for cannabinoids is needed to help orthopaedic traumatologists provide guidance for patients seeking advice for this recently popular therapeutic.

Cannabinoid compounds have been used for medicinal purposes for over 5 millennia.^1^ Despite a prolonged absence from routine medical conversations, patients and practitioners are now faced with increasing availability and exposure to cannabinoid-containing compounds for the treatment of various conditions. Sales of cannabinoid products increased from $9 billion in 2017 to an expected $20 billion in 2020.^2^ The United States Food and Drug Administration has approved cannabinoid formulations for antinausea in patients undergoing chemotherapy, appetite stimulation in acquired immunodeficiency syndrome-related anorexia, and for use in the treatment of rare forms of childhood epilepsy.^3^ In addition, cannabinoids have been studied in the treatment of mood and sleep disorders, glaucoma, and pain related to cancer and neuropathy.^4^ Active research explores the therapeutic benefits of cannabinoids in orthopaedics, particularly for back pain, hip and knee arthritis, trauma-related pain, and postsurgical pain.^5,6^

Cannabinoids are naturally occurring compounds in the *Cannabis sativa* plant, which is more commonly known as marijuana. Cannabinoids act through the endocannabinoid system, which is a lipid-signaling system involved in maintenance of physiologic homeostasis.^7,8^ The most well-known cannabinoids are tetrahydrocannabinol (THC) and cannabidiol (CBD). THC is a cannabinoid found in the cannabis plant; it is responsible for the psychoactive effects of cannabis. CBD is also a cannabinoid found in the cannabis plant. However, it has no psychoactive effects; instead, it has analgesic, anti-inflammatory, and anticonvulsant effects. Cannabinoids bind cannabinoid receptors (CB_1_ and CB_2_) and affect appetite, mood, and pain.^9^ The CB_1_ receptor is expressed throughout the body but has the highest concentration within the central nervous system, particularly in the nociceptive centers of the dorsal root ganglion. CB_1_ receptor abundance in nociceptive centers has led to research into how cannabinoids possibly play a role in the modulation of pain. Conversely, the CB_2_ receptor is primarily found within the immune system and, therefore, is believed to be involved in the regulation of inflammatory processes.^10^ Recent investigations have found wide expression of cannabinoid receptors in osseous tissue and evidence of positive cannabinoid impact on osteoarthritis disease progression, cartilage formation, and bone density.^11,12,13,14,15,16,17^

Despite increasing public enthusiasm, political support, and research in the medical applications of cannabinoids, a stigma regarding their use persists in both patients and practitioners. The sources of this stigma are myriad, but minimal research has been conducted regarding the current knowledge and sentiments that medical professionals have regarding cannabinoids.^18^ In the context of the continued opioid epidemic, the use of cannabinoids as a means of treating musculoskeletal pain and as a potential alternative to opioids has been an area of interest. As it stands currently, several factors (eg, sentiments among the medical community, stigmatization regarding the use of cannabinoids, and legislation) likely contribute to the lack of higher-quality studies into the use of cannabinoids within the field of orthopaedics. The purpose of this survey study was therefore to elucidate the sentiment and knowledge of cannabinoids within the orthopaedic trauma community.

## Methods

A 21-question survey (see Table, Supplemental Digital Content 1, http://links.lww.com/JG9/A123, which lists all questions of the survey and the order in which they appeared) was prepared and reviewed by the 3 senior authors (A.N., P.K.L, and J.A.S.). The anonymous survey included demographics questions and knowledge and perspective on cannabinoid use in orthopaedics (see Table, Supplemental Digital Content 1, http://links.lww.com/JG9/A123, which lists all questions of the survey). The questions were transferred onto an online survey software system, Qualtrics, which was also used to collect the survey data. The survey and proposal were sent to the Orthopaedic Trauma Association (OTA) Research Committee. After approval, the survey was posted on the OTA website for a three-month period. Electronic mail was sent by the OTA to all members soliciting participation in all posted surveys. Finally, direct communication by the senior authors (P.K.L and J.A.S.) to known peers within the orthopaedic trauma community was also performed to maximize the response rate. Survey participation was voluntary. Survey responses were collected. Microsoft Excel was used for tabulation of the survey data and statistical analysis.

## Results

### Demographics

In total, 251 (16.8%) of 1494 total active and clinical OTA members completed the survey. The demographics of the respondents were summarized (see Table, Supplemental Digital Content 2, http://links.lww.com/JG9/A124, which summarizes the demographics of the respondents). Most respondents (70%) practiced in an academic setting. More than half of all respondents (59%) were aged between 39 and 49 years; all age ranges (30-60 years and above) were represented. Almost all respondents (90.4%) practiced in states with some form of legalized cannabinoids. More specifically, 33.9% of respondents were from states with legalized medical and recreational cannabis, 36.7% were from states that had legalized only medical cannabis, and 19.9% practiced in states that had legalized only CBD or low-concentration THC formulations. Only 2.8% of respondents were from states without any legalized form of cannabis. Six percent of respondents did not identify the state where they practiced, and 0.8% of respondents were non-US residents.

### Orthopaedic Trauma Surgeons' Knowledge of Cannabinoids

**Figure 1:**
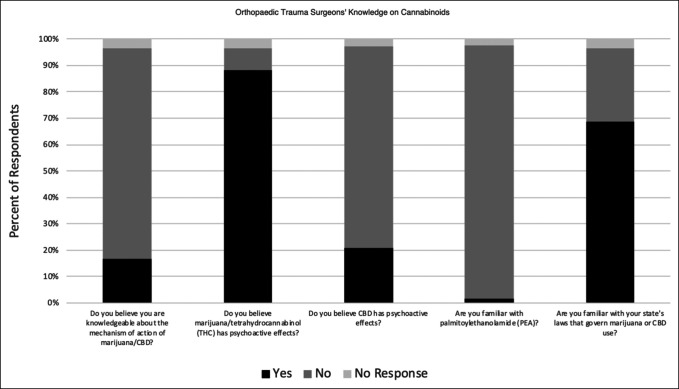
Bar graph showing survey responses that highlight surgeons' knowledge of cannabinoids. CBD = cannabidiol, THC = tetrahydrocannabinol, PEA = palmitoylethanolamide

The survey contained five questions to evaluate respondents' knowledge of cannabinoids. The distribution of the responses is summarized in Figure 1. Eighty-three percent of responding orthopaedic traumatologists did not believe that they were knowledgeable about the mechanism of action of cannabis/CBD. The fact that THC has psychoactive effects was correctly understood by 88% of respondents. Seventy-nine percent were correct in their understanding that CBD did not have psychoactive effects. More than 98% of respondents were not familiar with palmitoylethanolamide (PEA), an endocannabinoid currently being explored for its possible role in mitigating the inflammatory process. Regarding legal status, 68% were familiar with their state's laws governing marijuana or CBD use. The responses to questions relating to knowledge on cannabinoids are further delineated by state legalization status (Figure 3) and age group (Figure 4). No statistically significant difference was found in knowledge based on respondents' age group or state legal status of cannabis.

**Figure 2:**
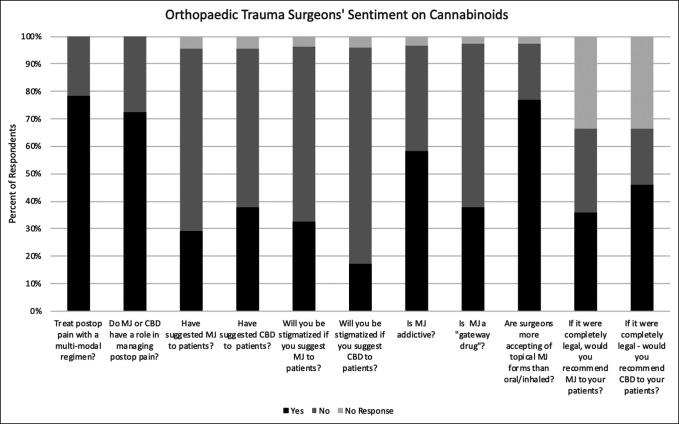
Bar graph showing survey responses that highlight surgeons' sentiments on cannabinoids. CBD = cannabidiol, MJ = marijuana, postop = postoperative

**Figure 3:**
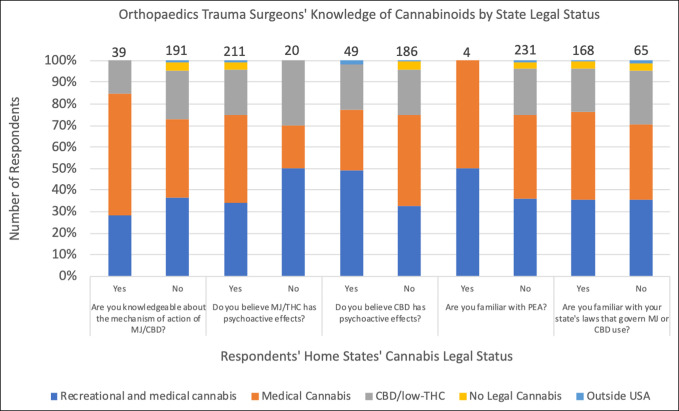
Bar graph showing survey responses that highlight surgeons' knowledge of cannabinoids by state legal status. CBD = cannabidiol, MJ = marijuana, PEA = palmitoylethanolamide, THC = tetrahydrocannabinol

**Figure 4:**
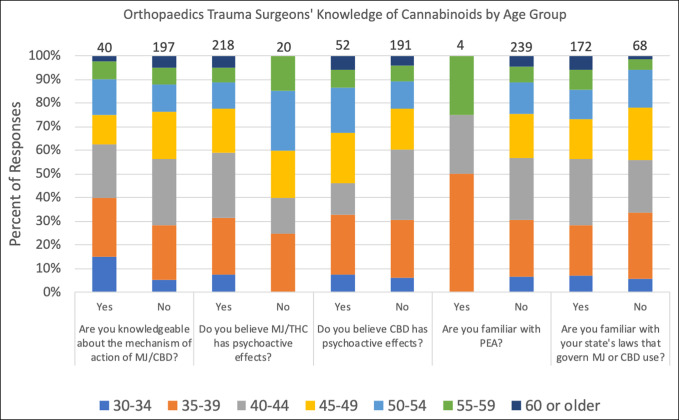
Bar graph showing survey responses that highlight surgeons' sentiments on cannabinoids by age group. CBD = cannabidiol, MJ = marijuana, PEA = palmitoylethanolamide, THC = tetrahydrocannabinol.

### Orthopaedic Trauma Surgeons' Sentiment on Cannabinoids

The survey contained 11 questions to evaluate respondents' sentiment on cannabinoids. The distribution of the responses is summarized in Figure 2. Notable findings are reported here. Multimodal postoperative pain regimens were used by 79% of respondents. Seventy-three percent of respondents believed that cannabis or CBD products play a role in management of postoperative pain. Cannabis had been suggested to patients by 29% of respondents. CBD had been suggested by 38% of respondents to their patients. Two-thirds of survey respondents did not believe that they would be stigmatized if they suggested cannabis to patients. Eighty-three percent of survey respondents did not believe that they would be stigmatized if they suggested CBD to patients. Cannabis was addictive in the opinion of 58% of respondents. Sixty-two percent do not believe that cannabis is a gateway drug. The belief that surgeons are more accepting of topical forms of cannabis than oral or inhaled forms was held by 77% of respondents. If it were completely legal (medical and recreational), 36% of respondents would recommend cannabis to patients. If it were completely legal (medical and recreational), 46% of respondents would recommend CBD to patients. Responses to questions about sentiment on cannabinoids were further delineated by state legal status (see Graph, Supplemental Digital Content 3, http://links.lww.com/JG9/A125, which delineates responses to questions related to cannabinoid sentiment by state legal status in a graph) and age group (see Graph, Supplemental Digital Content 4, http://links.lww.com/JG9/A126, which delineates responses to questions related to cannabinoid sentiment by age group in a graph).

Regarding whether a respondent has ever suggested cannabis to a patient, a statistically significant difference was observed between respondents from states with disparate legalization statuses (*P* = 0.009). The majority, regardless of state legalization statuses, have not suggested cannabis to patients as a means of treating postoperative pain. Regarding recommending CBD to patients if it were completely legal (medical and recreational), a statistically significant difference was observed between respondents from states with different legalization statuses (*P* << 0.01). Most respondents would recommend CBD if it were medically and recreationally legal. Regarding whether a respondent believed that cannabis or any CBD product has a role in the management of postoperative pain, the difference between respondents from different state legalization statuses was close to a statistically significant level (*P* ≈ 0.0507).

### Orthopaedic Trauma Surgeons' Sentiment on the Effects of Cannabinoids on Fracture Healing

The survey contained two questions to evaluate respondents' knowledge of the effects of cannabinoids on fracture healing. Regarding the effect of marijuana and CBD on fracture healing, 85% of respondents believed that no known effect exists. No statistically significant differences were found in fracture healing sentiments when separating out respondents by age group or state legalization status.

## Discussion

This survey study is the first and only time the orthopaedic surgery community has been assessed for its knowledge and opinion regarding cannabinoids. Our results provide the best available consensus on cannabinoids among practicing orthopaedic surgeons and allow them to understand how their peers are responding to the recent flood of cannabinoid products that are popular among patients, yet lack any endorsable research support.

Cannabis and its derivatives have become more mainstream, with a variety of products used by the young and the old, for recreation and for medicinal purposes.^19^ Medical professionals, including orthopaedic surgeons, are increasingly being approached for guidance on the use of these substances for analgesia and for the treatment of other musculoskeletal conditions. Although some research on cannabinoids regarding fracture healing,^20^ total knee arthroplasty,^21,22^ arthritis,^23^ and spine-related pain^24^ exists, high-quality evidence with respect to cannabis for management of musculoskeletal pain is scarce. The legal status of cannabinoids has been convoluted by regulatory differences between states and the federal government, whereas popular enthusiasm and anecdotal evidence is far outpacing scientific data.^19^ Thus, orthopaedic surgeons have had to balance low-quality clinical evidence with their professional opinions to advise patients.^5^

According to our survey results, the interest in cannabinoid products among orthopaedic trauma surgeons seems to mirror that of the public in terms of knowledge and sentiment. Many of the respondents believe in the existence of a role for cannabis or CBD in managing postoperative pain; however, a majority of respondents were not confident with their knowledge of the mechanism of action of cannabis or CBD. Most were correctly informed about the respective psychoactive effects or lack thereof for cannabis and CBD, respectively. As this survey was of fracture specialists, nearly all respondents were in agreement with the belief that no known effect on fracture healing is associated with cannabis or CBD. Previous studies have found that patients using cannabinoid products were stigmatized,^25,26^ but most orthopaedic trauma surgeons who responded to this survey did not believe that they would be stigmatized for suggesting them as providers. In particular, respondents felt that even less stigma surrounds the suggestion of CBD products. Most respondents did believe that a topical form of cannabis would be more accepted than an oral or inhaled form. Despite these opinions, only a third of respondents have acted on this belief and suggested cannabis or CBD to patients. Not surprisingly, only two-thirds of respondents were familiar with their states' laws governing marijuana or CBD use as this scenario is rapidly changing. After the November 2020 elections, four more states legalized recreational cannabis: Arizona, Montana, New Jersey, and South Dakota. That is, only three states remain without any form of legalized cannabis in the United States.^27^ These findings suggest that although orthopaedic trauma surgeons seem to view cannabinoids positively, more clinical research and legal clarification is needed to keep pace with commercial development and public support of these products.

However, state and federal legal differences on cannabinoids pose a significant challenge to studying its effects because it means that the products available to consumers and researchers are not consistent in strain or dosage. Researchers in the United States are restricted to a limited number of cannabis strains and products controlled by the National Institute on Drug Abuse in their studies, whereas commercially, products and strains are growing exponentially.^28^ Therefore, research findings may not be directly translatable to commercial products. Our study notes significant interest in cannabinoids within the orthopaedic trauma community. However, without support from federal legislation and adequate research, consensus for the use of cannabinoids in the treatment of a variety of medical conditions will be incredibly difficult to attain.

In addition, this survey found that nearly all respondents were unfamiliar with PEA, which is an endogenous cannabinoid classified as a nutraceutical, or a food that has potential health or medicinal benefits.^29^ PEA has activity at both CB_1_ and CB_2_ receptors, although it is not itself a component of cannabis^29^ and possesses anti-inflammatory effects.^30-32^ Therefore, the endocannabinoid system could be positively manipulated via PEA without negotiating the legal restrictions surrounding cannabis.

Limitations of this study include an incomplete data set and selection bias. Although the percentage of respondents (16.8%) may not be exceptionally high, the total number of respondents (251) is greater than or on par with other survey studies recently published in the orthopaedic trauma literature.^33-35^ Although this survey had 251 respondents, not all participants fully completed the questions, electing to defer their answers for some questions. In particular, 84 respondents did not provide answers to the two questions about whether they would recommend cannabis or CBD to their patients if it were completely legal. Such a loss of responses for two particularly important questions about the sentiment of cannabinoids skews the data and reduces the impact of this specific question. Selection bias is plausible as only orthopaedic traumatologists were invited to participate in the survey. Furthermore, 70% of these orthopaedic traumatologists practice in an academic setting, potentially leading to an additional source of bias. Regardless of these limitations, the findings of this study are invaluable to understanding the knowledge and sentiment of orthopaedic traumatologists regarding cannabis.

## Conclusion

Attitudes toward cannabinoids among orthopaedic traumatologists are favorable; however, in-depth knowledge of cannabis' mechanism is admittedly poor. This inconsistency is likely due to the illegal federal status of cannabinoids, which has impeded needed clinical research as industry promotion seems to have outstripped medical evidence. However, orthopaedic traumatologists seem to be cautiously aware of this dissonance and have not capitulated to freely recommending cannabinoids without evidence. More clinical research for cannabinoids is needed to help orthopaedic traumatologists provide guidance to their patients seeking advice for this recently popular therapeutic.

## Supplementary Material

SUPPLEMENTARY MATERIAL
