# Supplementary material for: Knowledge and Opinion on Cannabinoids Among Orthopaedic Traumatologists
Source: J Am Acad Orthop Surg Glob Res Rev. 2021 Apr 19;5(4):e21.00047. doi: 10.5435/JAAOSGlobal-D-21-00047 (PMC8057750; doi:10.5435/JAAOSGlobal-D-21-00047)
Supplement: SUPPLEMENTARY MATERIAL [file jagrr-5-e21.00047-s001.docx]

**Supplemental Digital Content 1: Survey Questions**

| **Question Number** | **Question** |
| --- | --- |
| 1 | Do you treat post-operative pain with a multi-modal (3 or more pain medications) regimen? |
| 2 | Do you believe there is a role for marijuana or any cannabidiol (CBD) product in managing post-operative pain? |
| 3 | Do you believe you are knowledgeable about the mechanism of action of marijuana/CBD? |
| 4 | Do you believe marijuana/tetrahydrocannabinol (THC) has psychoactive effects? |
| 5 | Do you believe CBD has psychoactive effects? |
| 6 | Are you familiar with palmitoylethanolamide (PEA)? |
| 7 | Have you suggested marijuana to any of your patients? |
| 8 | Have you suggested CBD to any of your patients? |
| 9 | Are you familiar with your state's laws that govern marijuana or CBD use? |
| 10 | Do you believe you will be stigmatized if you suggest marijuana to patients? |
| 11 | Do you believe you will be stigmatized if you suggest CBD to patients? |
| 12 | Do you believe marijuana is addictive? |
| 13 | Do you believe marijuana is a "gateway drug"? |
| 14 | Do you believe surgeons are more accepting of topical marijuana forms as compared to oral/inhaled? |
| 15 | Do you believe marijuana affects fracture healing? |
| 16 | Do you believe CBD affects fracture healing? |
| 17 | How would you describe your practice setting? |
| 18 | In which state do you practice |
| 19 | If it were completely legal (medical and recreational) - would you recommend marijuana to your patients? |
| 20 | If it were completely legal (medical and recreational) - would you recommend CBD to your patients? |
| 21 | Please select your age group. |
| Abbreviations: CBD - cannabidiol, THC - tetrahydrocannabinol, PEA - palmitoylethanolamide | |
