# Supplementary material for: Knowledge and Opinion on Cannabinoids Among Orthopaedic Traumatologists
Source: J Am Acad Orthop Surg Glob Res Rev. 2021 Apr 19;5(4):e21.00047. doi: 10.5435/JAAOSGlobal-D-21-00047 (PMC8057750; doi:10.5435/JAAOSGlobal-D-21-00047)
Supplement: SUPPLEMENTARY MATERIAL [file jagrr-5-e21.00047-s002.docx]

**Supplemental Digital Content 2: Demographics**

| **Practice Setting, Age, and Cannabis Legalization Status of Survey Respondents’ State of Residence** | | |
| --- | --- | --- |
| **Practice Setting** | Academic | 173 (70%) |
|  | Private Practice | 50 (20.2%) |
|  | Other | 21 (8.5%) |
|  | Practice Setting Not Identified | 3 (1.2%) |
| **Age** | 30-34 | 16 (6.6%) |
|  | 35-39 | 59 (24.2%) |
|  | 40-44 | 64 (26.2%) |
|  | 45-49 | 44 (18%) |
|  | 50-54 | 32 (13.1%) |
|  | 55-59 | 18 (7.4%) |
|  | 60 or older | 11 (4.5%) |
| **State of Residence** | Legalized and Recreational and Medical Cannabis | 85 (33.9%) |
|  | Legalized Medical Cannabis | 92 (36.7%) |
|  | Legalized CBD/low THC levels | 50 (19.9%) |
|  | No Legalized Cannabis | 7 (2.8%) |
|  | State of Residence Not Identified | 15 (6.0%) |
|  | Non-United States Resident | 2 (0.8%) |
| Abbreviations: CBD - cannabidiol, THC - tetrahydrocannabinol | | |
