# Supplementary material for: Knowledge and Opinion on Cannabinoids Among Orthopaedic Traumatologists
Source: J Am Acad Orthop Surg Glob Res Rev. 2021 Apr 19;5(4):e21.00047. doi: 10.5435/JAAOSGlobal-D-21-00047 (PMC8057750; doi:10.5435/JAAOSGlobal-D-21-00047)

**Supplemental Digital Content 3:** **Orthopaedics Trauma Surgeons' Sentiment on Cannabinoids by State Legal Status**


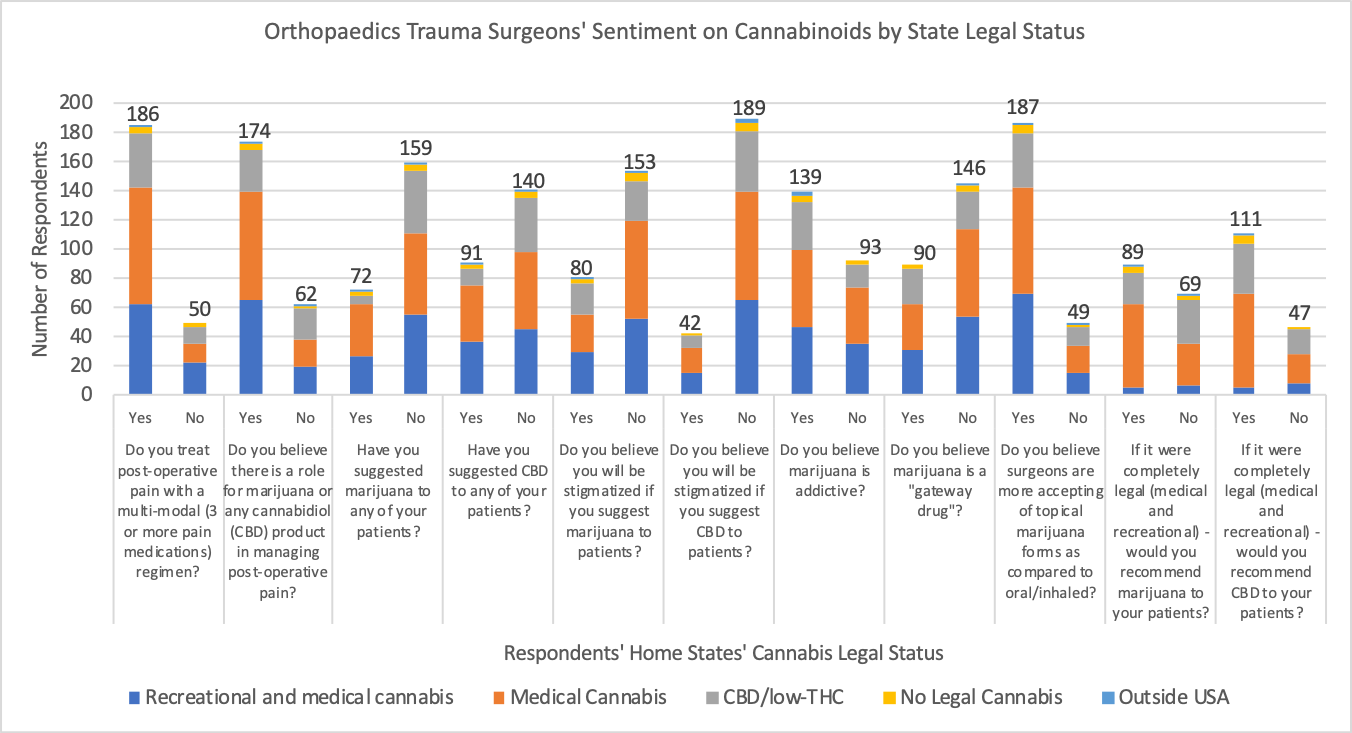

Supplement: SUPPLEMENTARY MATERIAL [file jagrr-5-e21.00047-s003.docx]
